# Stump Cholecystitis: Laparoscopic Completion Cholecystectomy with Basic Laparoscopic Equipment in a Resource Poor Setting

**DOI:** 10.1155/2014/787631

**Published:** 2014-08-21

**Authors:** Shamir O. Cawich, Carlos Wilson, Lindberg K. Simpson, Akil J. Baker

**Affiliations:** ^1^Department of Clinical Surgical Sciences, University of the West Indies, St. Augustine, Trinidad and Tobago; ^2^Department of Surgery, Percy Junor Hospital, Spalding, Jamaica; ^3^Department of Surgery, Kingston Public Hospital, Kingston, Jamaica; ^4^Department of Surgery, Mandeville Public Hospital, Manchester, Jamaica

## Abstract

*Introduction*. Stump cholecystitis is a recognised condition in which a large gallbladder remnant becomes inflamed after subtotal cholecystectomy. When this occurs, a completion cholecystectomy is indicated. Traditionally, these patients were subjected to open surgery because the laparoscopic approach was anticipated to be technically difficult. We present a case of completion cholecystectomy using basic laparoscopic equipment in a resource poor setting to demonstrate that the laparoscopic approach is feasible. *Case Description*. A 57-year-old woman presented with right upper quadrant pain and vomiting. She had an elective open cholecystectomy seven years before but reported remarkably similar symptoms. Abdominal ultrasound suggested calculous acute cholecystitis. MRCP confirmed the presence of a large gallbladder remnant with stones. Gastroduodenoscopy excluded other differentials. She had an uneventful laparoscopic completion cholecystectomy performed. *Discussion*. Although traditional dogma suggested that a completion cholecystectomy should be performed through the open approach, several small studies have demonstrated that laparoscopic completion cholecystectomy is feasible and safe. This report adds to the existing data in support of the laparoscopic approach.

## 1. Introduction

After a partial or subtotal cholecystectomy, symptoms may recur from pathology in the gallbladder remnant. When this occurs, a completion cholecystectomy is required to prevent a recurrence [1]. Many of these patients were traditionally subjected to open cholecystectomy because the laparoscopic approach was anticipated to be technically challenging. We present a case of a patient with stump cholecystitis in a gallbladder remnant to demonstrate that a laparoscopic approach to completion cholecystectomy is feasible using basic equipment in a resource poor setting.

## 2. Case Description

A 57-year-old woman with diabetes and hypertension presented to our hospital complaining of severe right upper quadrant pain and vomiting. She reported having an elective open cholecystectomy done seven years before and noted that the symptoms she now experienced were similar. She had been hospitalized six times in the preceding two years for similar episodes. Although abdominal ultrasounds were done on these occasions, the emergency room physicians did not entertain the possibility of a stump cholecystitis because the patient insisted that her gallbladder had already been removed. Operative notes were not available but she reported that the operation was uncomplicated and she was discharged 72 hours after open surgery.

Upper gastrointestinal studies excluded the presence of upper gastrointestinal pathology. Liver function panel and serum amylase levels were normal. Abdominal ultrasound suggested that stones were present in a thick walled gallbladder.

**Figure 1:**
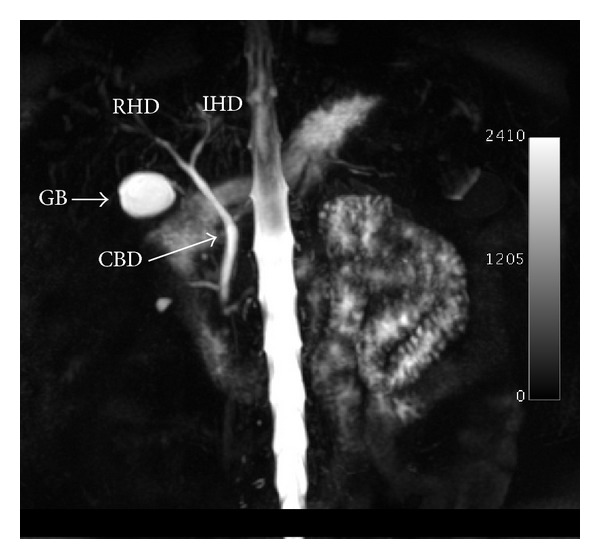
MRCP showing a normal extrahepatic biliary tree and a large gallbladder remnant (GB). CBD: common bile duct; LHD: left hepatic duct; RHD: right hepatic duct.

The patient's history was revisited on this presentation. She insisted that open cholecystectomy was performed seven years before and there was a surgical scar at the right upper quadrant, but no operative records or histology reports were available to corroborate the surgical history. Once the symptomatology resolved, the patient was discharged with a request for magnetic resonance cholangiography (MRCP) from another institution. This confirmed the presence of a gallbladder remnant with normal biliary anatomy. There was no evidence of choledocholithiasis (Figure 1).

**Figure 2:**
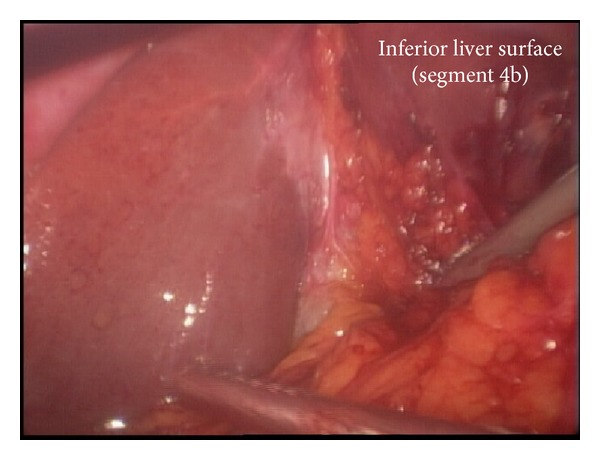
Dense adhesions occupy the gallbladder bed precluding clear visualization of anatomy.

**Figure 3:**
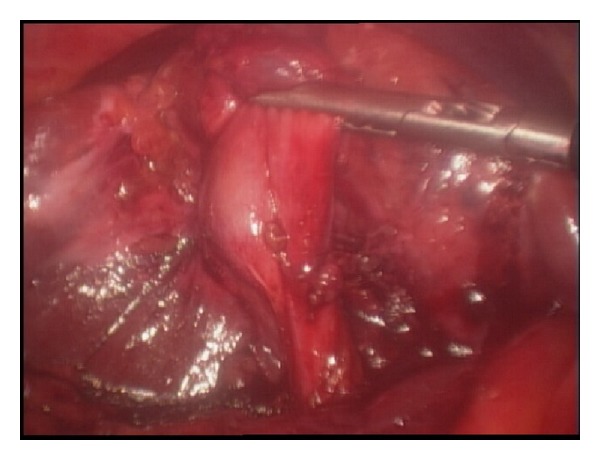
Careful dissection has presented the gallbladder remnant.

**Figure 4:**
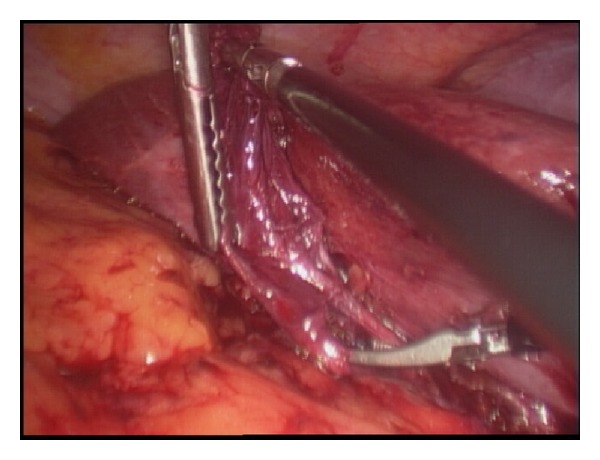
Anterograde dissection down to infundibulum presents structures in Calot's triangle and allows demonstration of Strasberg's critical view of safety.

**Figure 5:**
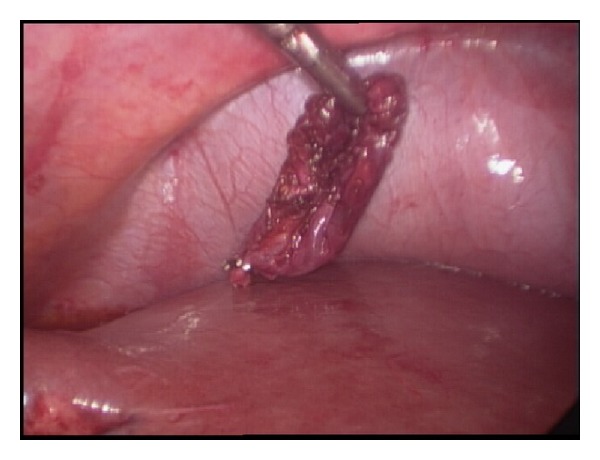
Cystic duct and artery divided and gallbladder remnant separated completely.

She was prepared for general anaesthesia and taken to the operating theatre for laparoscopic completion cholecystectomy. Laparoscopic shears were used to perform adhesiolysis in order to visualize the structures in the right upper quadrant. There were dense adhesions at gallbladder bed precluding clear visualization of relevant anatomy (Figure 2). Careful and patient dissection with the cautery hook eventually presented the gallbladder remnant (Figure 3). The remnant was followed in an anterograde fashion down to the cystic duct to demonstrate Strasberg's critical view of safety (Figure 4). The cystic duct and artery were then individually ligated and the gallbladder separated completely (Figure 5). Her recovery was uneventful and she was discharged home after 8 hours.

## 3. Discussion

A subtotal cholecystectomy is a safe option in the face of severe inflammation at Calot's triangle because it reduces the potential for common duct injury [2]. However, this carries the risk of developing stump cholecystitis when the gallbladder remnant becomes inflamed due to stone disease [1, 3].

The reported incidence of stump cholecystitis varies but has been reported to occur in as many as 5% [3] of patients after emergent cholecystectomy, and it is rare after elective operations. It tends to occur in middle-aged women who are usually quite confident that their symptoms are similar to those that prompted their original cholecystectomy [4]. Despite a suggestive history, the diagnosis is often delayed due to a low index of suspicion. Therefore clinicians should consider this diagnosis, especially after thorough investigations have excluded alternate pathologies.

Once the diagnosis is confirmed, the definitive treatment is a reoperation to excise the gallbladder remnant at a completion cholecystectomy [1, 3, 4]. These are technically difficult operations because there is usually significant scarring and anatomic distortion at the gallbladder bed [1, 3, 4]. Because a laparoscopic approach was thought to be unsafe in this circumstance, many patients were traditionally subjected to open surgery.

Gurel et al. [5] reported the first laparoscopic completion cholecystectomy in 1995. Since then, individual case reports [1, 3] and small case series [4] of laparoscopic completion cholecystectomies emerged once it was accepted that these patients also reap the benefits of minimally invasive surgery. These reports originated from developed countries, utilizing advanced technology such as ultrasonic dissection and high definition systems. This case was performed in a developing country with significant resource limitations using basic laparoscopic equipment that included laparoscopic graspers, scissors, and hook electrocautery. This adds to the existing data showing that the laparoscopic approach to completion cholecystectomy is feasible, even with basic equipment in the resource-poor setting.

## 4. Conclusion

Clinicians should maintain a high index of suspicion for the postcholecystectomy syndrome when patients present with a classic history. A laparoscopic approach to reoperative completion cholecystectomy is feasible even in the resource-poor setting.
